# Work Demands-Burnout and Job Engagement-Job Satisfaction Relationships: Teamwork as a Mediator and Moderator

**DOI:** 10.3889/oamjms.2015.024

**Published:** 2015-02-13

**Authors:** Dragan Mijakoski, Jovanka Karadzinska-Bislimovska, Vera Basarovska, Jordan Minov, Sasho Stoleski, Nada Angeleska, Aneta Atanasovska

**Affiliations:** *Institute for Occupational Health of Republic of Macedonia - Skopje, WHO Collaborating Center, Ga2len Collaborating Center, Skopje, Republic of Macedonia*

**Keywords:** Teamwork, work demands, burnout, job engagement, job satisfaction

## Abstract

**BACKGROUND:**

Few studies have examined teamwork as mediator and moderator of work demands-burnout and job engagement-job satisfaction relationships in healthcare workers (HCWs) in South-East Europe.

**AIM:**

To assess mediation and moderation effect of teamwork on the relationship between independent (work demands or job engagement) and dependent (burnout or job satisfaction) variables.

**METHODS:**

Work demands, burnout, job engagement, and job satisfaction were measured with Hospital Experience Scale, Maslach Burnout Inventory, Utrecht Work Engagement Scale, and Job Satisfaction Survey, respectively. Hospital Survey on Patient Safety Culture was used for assessment of teamwork. In order to examine role of teamwork as a mediating variable we fit series of regression models for burnout and job satisfaction. We also fit regression models predicting outcome (burnout or job satisfaction) from predictor (work demands or job engagement) and moderator (teamwork) variable.

**RESULTS:**

Teamwork was partial mediator of work demands-burnout relationship and full mediator of job engagement-job satisfaction relationship. We found that only job engagement-job satisfaction relationship was moderated by teamwork.

**CONCLUSIONS:**

Occupational health services should target detection of burnout in HCWs and implementation of organizational interventions in hospitals, taking into account findings that teamwork predicted reduced burnout and higher job satisfaction.

## Introduction

Health care workers (HCWs) as the key actors of the health care system provide health services to the patients that should be safe, effective, patient-centred, timely, efficient, and equitable [1, 2]. Hospital HCWs are exposed to different hazards at the workplace including psychosocial hazards that originate from the workplace demands and conditions [3, 4]. The hospital context characterized by wide range of hazards and risks creates conditions for development of work-related stress as a harmful physical and emotional response in circumstances when requirements of the work do not match the capabilities, resources or needs of the worker [5].

Exposure to chronic emotional and interpersonal workplace stressors can lead to burnout syndrome [6-8]. Hospital settings encompass different physical, psychological, social, or organizational factors that require prolonged physical and/or psychological efforts in workers. These factors are referred as work demands. They are not necessarily negative, but they may turn into workplace stressors when the invested personal efforts are high. In such circumstances, work demands are associated with certain physiological and/or psychological costs [9]. Respectively, high work demands are leading to overtaxing and emotional exhaustion. On the other hand, the lack of job resources results in withdrawal behavior or depersonalization and disengagement [8, 10, 11].

Job resources are defined as those aspects of the job that reduce work demands and the associated costs, and stimulate personal growth, learning and development [9]. In the context of reduced job resources (e.g., inappropriate performance feedback, low salary, job insecurity, inadequate supervisory coaching and teamwork), work demands are particularly effective and detrimental [10-12].

According to the Job Demands-Resources model of burnout (JD-R Model) [11], an energetic process of overtaxing involves high job demands that exhaust the worker’s energy (emotional exhaustion) as well as downward adjustment of performance targets (cynical attitudes towards work, depersonalization, and disengagement) [13]. Additionally, a motivational process encompasses lack of recourses that buffer dealing effectively with high job demands and emphasize mental withdrawal or disengagement [9]. Certain job resources are linked with high-quality work performance, low absenteeism and turnover, and job satisfaction [14].

Teamwork refers to specific cooperative process that allows team members to develop effective, mutual relationships in achieving team goals through sharing knowledge and skills [15, 16]. Teamwork is critical for the delivery of health care services and HCWs must coordinate their activities to deliver safe and efficient patient care [17]. In addition, teamwork as a particular job resource, protect workers from emotional exhaustion, depersonalization, and disengagement [9].

Work contexts with many job resources (e.g., supportive colleagues, teamwork, and proper feedback from superiors) increase the willingness of workers’ to dedicate their efforts to the work tasks. In such conditions, a positive outcome can be found with engaged workers who are satisfied with their job and have a low tendency to leave the organization.

Job engagement is defined as a positive work-related state of mind and originally considered to be at the opposite pole of burnout [12]. The core components of job engagement are vigour (high levels of energy and mental resilience while working) and dedication (sense of significance, enthusiasm, inspiration, pride, and challenge) [18]. However, Schaufeli and Bakker view job engagement as a separate phenomenon that can function independently of burnout [19] and assume the existence of two processes: energetic process driven by work demands and workers’ efforts in which burnout plays a key role leading to poor health, and motivational process driven by available job resources in which job engagement has a central role resulting in workers who are satisfied with their job [9]. Within organizational context, job satisfaction refers to the extent to which work is a source of need fulfillment and contentment and this component of the work life keeps people job involving [20].

Scientific literature clearly shows that burnout is linked with high job demands [21]. On the other hand, Saks showed that job engagement is related to workers’ attitudes (i.e. job satisfaction), intentions, and behaviors [22]. The experience of engagement has been also found to be related to good health and positive work affect, such as job satisfaction [23]. It is well established that work organizations with proper feedback, adequate supervisor and coworker support, and appropriate teamwork demonstrate low depersonalization and high level of job engagement in their workers, while the absence of these specific job resources increases cynical attitudes towards work [10-12]. Furthermore, the importance of teamwork within health care settings in the promotion of job satisfaction, reduction of job demands, and its positive effects on job engagement are shown elsewhere [24-27].

Despite numerous studies on work-related stress and burnout in HCWs, to date very few studies have examined teamwork as a mediator and moderator of the relationships between work demands and burnout and between job engagement and job satisfaction in the region of South-East Europe (SEE). In this study we analyzed HCWs working in a surgery clinic in Skopje, Macedonia that is an educational base of the Faculty of Medicine in Skopje, providing health care to the general population at secondary and tertiary level.

The objectives of the actual study were to assess:

- the associations between work demands, burnout, job engagement, job satisfaction, and teamwork, controlling for age, hospital tenure, unit tenure, and working hours per week as potential confounders;

- the mediation effect of teamwork on the relationship between independent (work demands or job engagement) and dependent (burnout or job satisfaction) variables; and

- the moderation effect of teamwork on the relationship between predictor (work demands or job engagement) and outcome variables (burnout or job satisfaction).

## Methods

The study was conducted during September-December 2014. A self-administered questionnaire prefaced with an invitation letter and information about the study was sent to HCWs working in a surgery clinic, an educational base of the Faculty of Medicine in Skopje. Official approval was obtained by the Hospital Ethics Committee.

We used Hospital Experience Scale (HES) for the assessment of work demands. The HES was constructed and developed for the purposes of FP7 ORCAB Project (http://orcab.web.auth.gr/). Participants indicated their level of agreement with the items on physical workload (seven items), organisational (six items), emotional (six items) and cognitive (five items) work demands on a 5-point Likert scale (1 = never to 5 = always). In this study only the total score of work demands (α=0.89) was used, calculated as a mean score of the points for statements representing work demands. Higher mean score delineates higher perceived level of work demands.

Burnout was measured using the Maslach Burnout Inventory (MBI) [28]. Emotional exhaustion (nine items) and depersonalisation (five items) were assessed with a 7-point Likert scale (0 = never to 6 every day). In this study we used the total score of burnout (α = 0.89), calculated as a sum of both burnout dimensions.

Job engagement was assessed with the Utrecht Work Engagement Scale (UWES) [18]. Vigor (six items) and dedication (five items) were measured with a 7-point Likert scale (0 = never to 6 = every day). The total score of job engagement (α = 0.93) was used as a sum of vigour and dedication.

We used Job Satisfaction Survey (JSS) [29] for the measurement of job satisfaction. The items of the JSS instrument (39 items) represent seven job satisfaction factors: general attitudes (five items), and planning (five items), performance (six items), management (five items), supervisory (six items), training and salary (five items), and benefits issues (seven items). Participants indicated level of agreement with the items (1 = strongly agree, 2 = agree, 3 = neither agree nor disagree, 4 = disagree, 5 = strongly disagree). Prior to analysis, we re-coded the answers on JSS items (1=5, 2=4, 3=3, 4=2, 5=1). The purpose was to get higher points for higher level of agreement with the statements and vice versa since all items were stated in a positive direction (ex., there is adequate planning of hospital objectives; I believe my job is secure). The total score of job satisfaction (α=0.96) was used in this study and it was calculated as a mean score of the points for statements representing job satisfaction. Higher mean score represents higher level of perceived job satisfaction.

Teamwork (four items, α = 0.85) was measured with the Hospital Survey on Patient Safety Culture (http://www.ahrq.gov/qual/patientsafetyculture/hospcult1.htm). Participants indicated level of agreement with the items (1 = strongly disagree, 2 = disagree, 3 = neither agree nor disagree, 4 = agree, 5 = strongly agree) and the mean score was calculated. Higher mean score delineates higher level of perceived teamwork.

Completed surveys were returned by 151 health care workers - 40 physicians (specialists, interns, and residents), 71 nurses/technicians, and 40 nursing aids - working in a surgery clinic. Participants were 68.2% female (N = 103) and they had an average age of 44.73 (SD = 9.66) years. They worked for an average of 198.85 (SD = 140.09) months at the same hospital, for 128.62 (SD = 126.51) months within the same unit, and for 41 hours per week (SD = 5.06).

### Analysis

Initially, bivariate analyses were conducted to examine the associations between work demands, burnout, job engagement, job satisfaction, and teamwork. Secondly, in order to assess the role of work demands (for burnout), job engagement (for job satisfaction), and teamwork, controlling for age, hospital tenure, unit tenure, and working hours per week, separate hierarchical multiple regression models were tested for both burnout and job satisfaction. Age, hospital tenure, unit tenure, and working hours per week were entered in the first step, work demands (or job engagement) were entered in the second step, while teamwork was entered in the third step.

Additionally, we fit regression models analysing teamwork as a mediating variable and its significance in the models. A series of regression models were fitted for both burnout and job satisfaction: model 1 predicting the mediator variable (teamwork) using the independent variable (work demands or job engagement); model 2 predicting the dependent variable (burnout or job satisfaction) using both the independent variable and the mediator; and model 3 predicting dependent variable by using the independent variable.

Aforementioned models were used to test following conditions of mediation:

- the independent variable must significantly predict the dependent variable in model 3;

- the independent variable must significantly predict the mediator in model 1;

- the mediator must significantly predict the dependent variable in model 2; and

- the independent variable must predict the dependent variable less strongly in model 2 than in model 3.

During this process, when we have confirmed the insignificance of the relationship between the independent variable and the dependent variable in the presence of the mediator (within model 2), we have defined that case as a case of full mediation of the relationship between independent variable and dependent variable. Otherwise, when we have confirmed that independent variable predicted the dependent variable less strongly in model 2 than in model 3, but the relationship between the independent variable and the dependent variable remained significant in the presence of the mediator (within model 2), we have defined that case as a case of partial mediation of the relationship between independent variable and dependent variable.

We also applied the Sobel test that produces a significance test of the indirect effect of independent variable on dependent variable through mediator. This test was used to assess whether a mediator carries the influence of an independent variable to a dependent variable. But, because bootstrapping offers more reliable results, we used both bootstrapping and Sobel test during mediation analysis.

Finally, we fit regression models predicting the outcome variable (burnout or job satisfaction) from both the predictor variable (work demands or job engagement) and the moderator variable (teamwork). We add the interaction effects (of both the predictor variable and the moderator variable) to the previous models and check for a significant *R*^2^ change as well as significant effects by the new interaction terms. The data obtained were used to produce interaction plots for visualizing conditional (moderated) effect of predictor (independent variable) on the outcome (dependent variable).

## Results

The mean score for work demands was 2.88 (SD = 0.58) (on the scale from 1 = never to 5 = always). The mean scores for emotional exhaustion, depersonalization, and total burnout were 18.99 (SD = 12.47), 3.68 (SD = 4.55), and 22.66 (SD = 15.17), respectively. The average scores for vigour, dedication, and total job engagement were 26.51 (SD= 9.46), 22.8 (SD = 7.96), and 48.85 (SD = 17.12), respectively. Study subjects demonstrated relatively high overall satisfaction with their job - 3.43 (SD = 0.71) (on the re-coded scale from 1 = strongly disagree to 5 = strongly agree) and perceived relatively high level of teamwork within their unit - 3.89 (SD = 0.75) (1 = strongly disagree to 5 = strongly agree).

In this study work demands score was positively correlated with total burnout score. Total job engagement score was also positively correlated with job satisfaction score. Teamwork was negatively correlated with burnout and positively correlated with job satisfaction. There was significant negative correlation between teamwork and work demands, and significant positive correlation between teamwork and job engagement.

Standardized beta coefficients for the independent predictors of burnout in study sample are shown in Table 1. Work demands (*β* = 0.41, *p* < 0.001) positively predicted burnout. On the other hand, teamwork (*β* = -0.25, *p* < 0.05) was significant negative predictor of burnout (*R*^2^ for the model = 0.349).

**Table 1:** Hierarchical multiple regression model for burnout in study sample.

Burnout		B	SE	95% CI for B		Beta	*R*^2^
**Step 1**				**Lower**	**Upper**		0.035

	Age	0.08	0.26	-0.44	0.61	0.05	

	Hospital Tenure	0.01	0.02	-0.03	0.05	0.10	

	Unit Tenure	-0.01	0.02	-0.05	0.03	-0.08	

	Working hours per week	0.49	0.31	-0.14	1.11	0.16	

	Constant	-3.26	15.39	-33.82	27.29		

**Step 2**	Age	0.05	0.23	-0.39	0.50	0.03	0.302

	Hospital Tenure	0.02	0.02	-0.01	0.06	0.17	

	Unit Tenure	-0.02	0.02	-0.05	0.02	-0.11	

	Working hours per week	0.39	0.27	-0.14	0.93	0.13	

	**Work Demands**	**13.01**	**2.17**	**8.70**	**17.32**	0.52**	

	Constant	-36.35	14.27	-64.69	-8.02		

**Step 3**	Age	0.08	0.22	-0.35	0.52	0.05	0.349

	Hospital Tenure	0.02	0.02	-0.02	0.05	0.15	

	Unit Tenure	-0.01	0.02	-0.04	0.02	-0.09	

	Working hours per week	0.32	0.26	-0.21	0.84	0.10	

	**Work Demands**	**10.33**	**2.35**	**5.66**	**14.99**	0.41**	

	**Teamwork**	**-4.84**	**1.87**	**-8.55**	**-1.13**	-0.25*	

	Constant	-7.28	17.83	-42.68	28.12		

*R*^2^ = .035 for Step 1; Δ *R*^2^ = .267 for Step 2 (*P*<0.001); Δ *R*^2^ = .047 for Step 3 (*P*<0.05)

* *p*<0.05,

** *p*<0.001.

Table 2 demonstrates that teamwork (*β* = 0.64, *p* < 0.001) was the sole positive predictor of job satisfaction (*R*^2^ for the model = 0.459).

**Table 2:** Hierarchical multiple regression model for job satisfaction in study sample.

Job satisfaction		B	SE	95% CI for B		Beta	*R*^2^
**Step 1**				**Lower**	**Upper**		0.017

	Age	0.01	0.01	-0.01	0.03	0.12	

	Hospital Tenure	1.73E-6	0.001	-0.002	0.002	0.00	

	Unit Tenure	0.000	0.001	-0.002	0.001	-0.05	

	Working hours per week	-0.01	0.01	-0.04	0.02	-0.08	

	Constant	3.69	0.70	2.31	5.07		

**Step 2**	Age	0.01	0.01	-0.01	0.03	0.13	0.114

	Hospital Tenure	2.61E-5	0.001	-0.002	0.002	0.01	

	Unit Tenure	-9.59E-5	0.001	-0.002	0.001	-0.02	

	Working hours per week	-0.02	0.01	-0.05	0.01	-0.12	

	**Job engagement**	**0.02**	**0.004**	**0.01**	**0.02**	0.32*	

	Constant	3.08	0.69	1.72	4.45		

**Step 3**	Age	0.01	0.01	-0.01	0.02	0.09	0.459

	Hospital Tenure	3.86E-5	0.001	-0.001	0.001	0.01	

	Unit Tenure	0.000	0.001	-0.001	0.001	-0.05	

	Working hours per week	-0.01	0.01	-0.03	0.02	-0.03	

	Job engagement	0.004	0.004	-0.003	0.01	0.08	

	**Teamwork**	**0.61**	**0.07**	**0.46**	**0.76**	0.64**	

	Constant	0.86	0.60	-0.34	2.05		

*R*^2^ = .017 for Step 1; Δ *R*^2^ = .097 for Step 2 (*P*<0.001); Δ *R*^2^ = .345 for Step 3 (*P*<0.05)

* *p*<0.01,

** *p*<0.001.

Regression model analysing teamwork as a mediating variable demonstrated that there was a significant indirect effect of work demands on burnout through teamwork, *b* = 2.319, *BC*α CI (0.576, 5.003). This represents medium effect, *k*^2^ = 0.09, 95% *BC*α CI (0.024, 0.194). Overall analysis and Sobel test showed partial mediation in the model (*z* = 2.334, *p* = 0.02). It was detected that teamwork partially mediated the relationship between work demands and burnout.

The study also demonstrated significant indirect effect of job engagement on job satisfaction through teamwork, *b* = 0.006, *BC*α CI (0.002, 0.011) and this represents relatively large effect, *k*^2^ = 0.14, 95% *BC*α CI (0.055, 0.236). Mediation analysis using bootstrapping and Sobel test demonstrated full mediation in the model (*z* = 2.73, *p* = 0.006) showing that teamwork fully mediated the relationship between job engagement and job satisfaction.

Results of the moderation analysis for the relationship between work demands and burnout using teamwork as a moderator are shown in table 3. It is demonstrated that the interaction between teamwork and work demands is not significant, *b* = 1.36, 95% CI (-2.33, 5.04), *t* = 0.73, *p* = 0.468, indicating that the relationship between work demands and burnout is not moderated by teamwork.

**Table 3:** Results of the moderation analysis for the relationship between work demands and burnout using teamwork as a moderator.

	*b* (95% CI)	*SE B*	*t*	*p*
Teamwork	-4.86 (-8.20, -1.53)	1.69	-2.88	0.005

Work demands	11.40 (6.76, 16.03)	2.34	4.86	<0.001

Teamwork x Work demands	1.36 (-2.33, 5.04)	1.86	0.73	0.468

Constant	22.09 (19.64, 24.54)	1.24	17.84	<0.001

*Note: R*^2^ = 0.303

Table 4 shows the results of the moderation analysis for the relationship between job engagement and job satisfaction using teamwork as a moderator. The interaction between teamwork and job engagement is highly significant, *b* = 0.01, 95% CI (0.003, 0.02), *t* = 2.69, *p* = 0.008, indicating that the relationship between job engagement and job satisfaction is moderated by teamwork.

**Table 4:** Results of the moderation analysis for the relationship between job engagement and job satisfaction using teamwork as a moderator.

	*b* (95% CI)	*SE B*	*t*	*P*
Teamwork	0.56 (0.42, 0.70)	0.07	8.07	<0.001

Job engagement	0.003 (-0.003, 0.01)	0.003	1.10	0.274

Teamwork x Job engagement	0.01 (0.003, 0.02)	0.004	2.69	0.008

Constant	3.44 (3.34, 3.54)	0.05	67.34	<0.001

*Note: R*^2^ = .372

**Figure 1:**
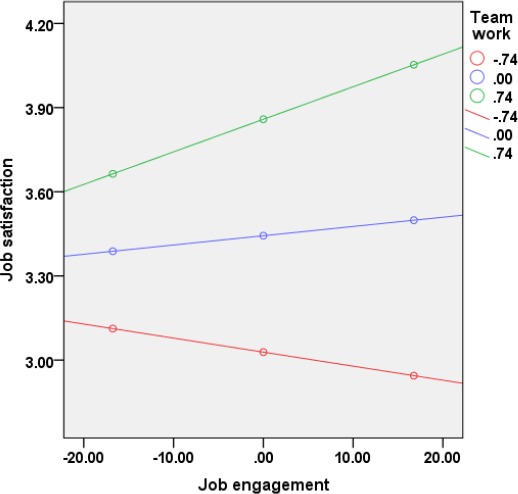
*Interaction plot showing enhancing effect of the teamwork on the development of job satisfaction via job engagement in study sample*.

Additionally, the examination of the interaction plot (Figure 1) showed an enhancing effect, where increasing the perception of teamwork would increase the effect of job engagement on the job satisfaction. In HCWs with lower job engagement, job satisfaction was similar for low, average, or high teamwork. HCWs with the highest job engagement and high perceived teamwork were the most satisfied with their job.

## Discussion

Emotional exhaustion (18.99) and depersonalisation (3.68) average scores in this study were lower than in previous studies on burnout in hospital HCWs [30]. On the other hand, the mean scores of vigour (26.51) and dedication (22.8) were similar or higher than those detected in other studies on job engagement in HCWs [31, 32]. According to the JD-R Model [10], work demands that showed medium level (2.9) as perceived by the participants in the actual study, together with relatively high level of perceived teamwork within hospital unit (3.9) (one of the most important job resources along with support from superiors, and independence in decision making), could boost the compensatory efforts in HCW in order to maintain high level of performance (high job engagement) and to reduce physiological and psychological costs in HCWs associated with their work efforts (low exhaustion and depersonalisation) [30]. Descriptive data obtained in this study also showed relatively high level of overall satisfaction with the job - 3.4, the mean score that together with the added value of the standard deviation - 0.7 becomes over 4, the level that is more than acceptable for job satisfaction.

Up to now, similar studies assessing teamwork as a mediator and moderator of the relationships between work demands and burnout and between job engagement and job satisfaction in hospital HCWs in the SEE Region are very rare.

Actual study demonstrated that work demands predicted higher burnout levels. The more frequently that HCWs experienced work demands (i.e., strict hierarchy in the hospital, problematic communication with hospital management, excessive workload, time pressure, lack of staff and supplies, a lot of competitiveness with the colleagues, emotional involvement in work), the more they felt burnt out. On the other hand, teamwork was related to lower levels of burnout. The more that HCWs experienced teamwork (e.g., support from colleagues, working together as a team, offering help to colleagues), the less they experienced burnout. Teamwork also predicted higher level of satisfaction with the job. The more that HCWs experienced teamwork, the more they were satisfied with the adequacy of planning, hospital conditions, job security, given recognition by the management, support by the superiors, ongoing training, salary, overall benefits, etc.

Similarly to our findings, Schaufeli and Bakker showed that burnout was predicted by high work demands and lack of job resources (i.e., lack of teamwork) [9]. In other study the same authors demonstrated that decreases in job resources (i.e., autonomy, teamwork, feedback) predict burnout [33]. It was also shown that the likelihoods of having higher burnout scores were lower in units with good quality of working relationships between nurses and physicians [30].

Hierarchical multiple regression analysis for job satisfaction in the study sample demonstrated that in the second step job engagement positively predicted job satisfaction. Similarly, the study of Orgambidez-Ramos et al. [34] suggested that engagement is a key predictor of job satisfaction. These findings are also consistent with the results from other studies [10, 22, 35, 36]. But, the entrance of teamwork in the model in the third step resulted in change of significant predictors of job satisfaction. The standardized beta coefficient of job engagement was no more significant, while teamwork became the sole predictor of job satisfaction. Contrary to that, work demands significantly predicted burnout both before and after entering the teamwork in the hierarchical multiple regression models for burnout.

Aforementioned observations encouraged us to fit regression models analysing teamwork as a mediating variable. We have found that teamwork was a significant partial mediator of the relationship between work demands and burnout. Higher levels of work demands were associated with lower levels of teamwork, which, in turn, were associated with increased burnout level. This shows that work demands were directly and indirectly related to burnout through teamwork. HCWs who perceived higher levels of work demands, also perceived lower teamwork within their unit and were therefore more burnt out. Maintaining low perceptions of work demands among HCWs would most likely result in higher perception of teamwork, which in turn will reduce the feelings of burnout. Similar findings were reported by Dubreuil et al. [37] showing that the relationships between workplace stressors and burnout were partially mediated by job resources (i.e., social relationships at work).

Additionally, actual study shows that teamwork was a significant full mediator of the relationship between job engagement and job satisfaction. Higher levels of job engagement were associated with higher levels of teamwork that, in turn, were associated with increased job satisfaction. Teamwork was also reported as an important factor for promotion of job satisfaction and job engagement in many other studies [24-27]. Our data emphasized that teamwork had larger mediating effect on the relationship between job engagement and job satisfaction than on the relationship between work demands and burnout.

Finally, we have found that in the study sample only the relationship between job engagement and job satisfaction was moderated by teamwork, while the relationship between work demands and burnout was not moderated by this job resource. In such context, teamwork showed an enhancing effect (i.e., increasing the teamwork would increase the effect of job engagement on the job satisfaction). The more that HCWs felt teamwork in their unit, the more that job engagement had an increasing effect on job satisfaction.

The results of mediation and moderation analyses clearly demonstrated that teamwork as a particular job resource had stronger effects on the relationship job engagement-job satisfaction than on the relationship work demands-burnout. These findings are in line with the theoretical and empirical data concerning the existence of two processes: 1. energetic process involving work demands - burnout - poor health, and 2. motivational process including job resources - job engagement - job satisfaction [9, 19] with job resources having weaker effect on the relationship work demands-burnout.

The actual study was cross-sectional, which limited our ability to make causal inferences. We included several confounding factors in the models, but it is possible that some were missed. Our findings may reflect a particular response bias because HCWs experiencing high levels of burnout may have lacked motivation to participate in the study. Also, a “healthy worker effect” may have under-estimated the levels of burnout in the total sample.

As a conclusion, this study shows that work demands were related to burnout in surgery clinic health care workers. On the other hand, perceived teamwork was related to job satisfaction and lower levels of burnout. Furthermore, it is shown that teamwork was a significant partial mediator of the relationship between work demands and burnout as well as significant full mediator of the relationship between job engagement and job satisfaction. Finally, we have found that only the relationship between job engagement and job satisfaction was moderated by teamwork. Given that, these findings support the idea of co-existence of energetic and motivational process and job resources (i.e., teamwork) have an effect that is of different size on the different relationships (work demands-burnout and job engagement-job satisfaction).

We found lower average emotional exhaustion and depersonalisation scores contrary to the existing burnout literature. Compensatory efforts in HCWs driven by work demands in order to maintain high level of performance (high engagement) and to reduce physiological and psychological costs in HCWs (low exhaustion and depersonalisation) could have an important role. In accordance with this, we found engagement scores (vigour and dedication) similar or higher than those detected in other studies.

Our major findings suggest that feelings of burnout in HCWs are highly influenced by the frequency of perceived work demands and feelings of teamwork in the hospital unit. Furthermore, teamwork highly influences the feelings of satisfaction with the job. Also, teamwork is detected as a mediator of the relationships between work demands and burnout and between job engagement and job satisfaction, as well as a moderator of job engagement-job satisfaction relationship. Occupational health services should target the early detection of burnout in HCWs, as well as the development and implementation of specific organizational interventions in hospital settings. These interventions should take into account the findings that teamwork predicted reduced burnout and higher job satisfaction. Aforementioned measures together with appropriate management of work demands can reduce the feelings of burnout and improve the satisfaction with the job in health care workers, as well as to improve quality of patient care.
